# Electrophysiological study and radiofrequency ablation of hemodynamically-instable ventricular arrhythmias in a patient with pulmonary hypertension: A case report

**DOI:** 10.1097/MD.0000000000024896

**Published:** 2021-02-26

**Authors:** Song Zou, Zhifu Cen, Ruikun Jia, Sijie Lu, Yan Hao, Kaijun Cui

**Affiliations:** a Department of Cardiology, West China Hospital; b State Key Laboratory of Biotherapy, Sichuan University, Chengdu, Sichuan, People's Republic of China.

**Keywords:** case report, enhanced cardiac CT, pulmonary hypertension, radiofrequency ablation, ventricular arrhythmias

## Abstract

**Introduction:**

Hemodynamically-instable ventricular arrhythmias (VAs) are rare in patients with pulmonary hypertension (PH). To the best of our knowledge, only 1 case has been reported so far. Moreover, the pathogenesis of this kind of arrhythmia remains obscured and its treatment is challenging. Here we report another case and presented the substrate for VAs initiation and therapeutic effect of radiofrequency ablation.

**Patient concerns:**

This is a 57-year-old man who presented paroxysmal palpitation associated with presyncope at rest. Surface electrocardiogram (ECG) revealed frequent ventricular premature contractions and non-sustained ventricular tachycardia when symptoms occurred. He also had a history of severe PH which was secondary to atrial septal defect and partial anomalous pulmonary venous drainage and suffered from obvious dyspnea when climbing stairs World Health Organization Class III (WHO Class III).

**Diagnosis:**

Hemodynamically-instable VAs associated with severe PH.

**Intervention:**

Echocardiography revealed enlargement of right ventricle (right ventricle [RV]: 43 mm). Electrophysiological examination showed the origin of VAs is next to a small low-voltage zone of RV. Radiofrequency delivery at the origin successfully terminated VAs without occurrence of complication.

**Outcome:**

The patient was free from arrhythmias and got an improvement of exercise tolerance, just with mild dyspnea when climbing stairs World Health Organization Class II (WHO class II), during six-month follow up.

**Lessons:**

This case suggests the low-voltage zone of remodeled RV, which may be secondary to increased pulmonary artery pressure, serves as the substrate for VAs initiation in patient with PH. Radiofrequency ablation can successfully terminate VAs and the termination of VAs can significantly improve the patient's impaired exercise tolerance.

## 1 Introduction

Although the dysfunction of right ventricle (RV) is the most common complication in patients with pulmonary hypertension (PH), the episode of hemodynamically-instable ventricular arrhythmias (VAs) in these patients is rare. Moreover, information about the electrophysiological characteristics, substrate and treatment of this kind of patients is lacking. Up to now, only 1 case has been reported.^[1]^ Herein, we reported another case whose electrophysiological study (EPS) demonstrated the substrate of VAs is a small low-voltage zone which is different from the previous one. Radiofrequency ablation (RFA) successfully terminated VAs and the patient got an improvement of exercise tolerance despite the exacerbation of PH.

## 2 Case presentation

**Figure 1:**
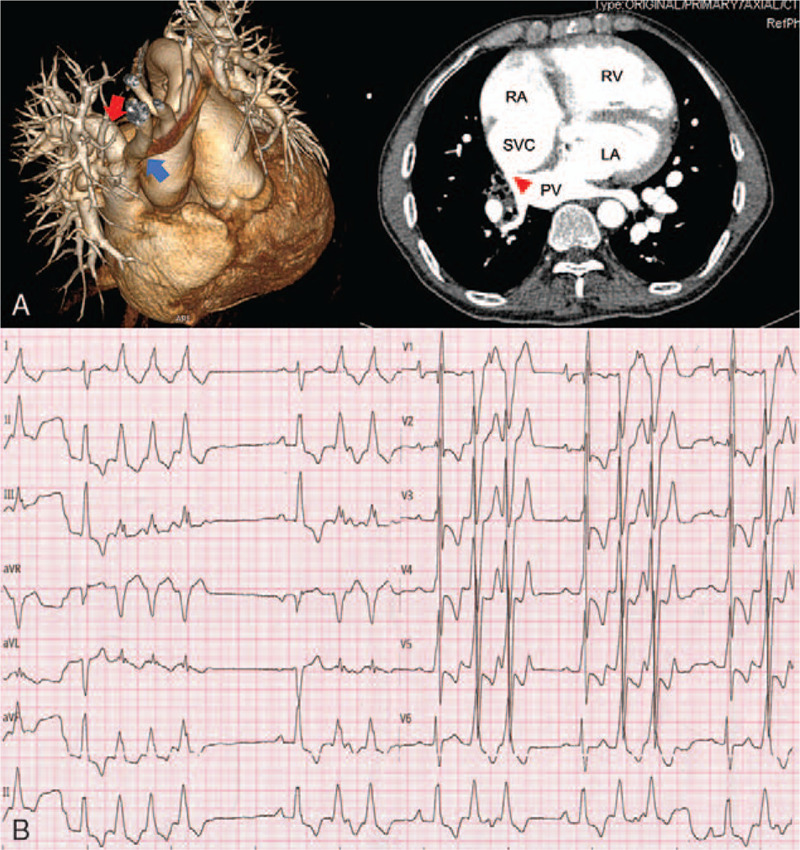
ECG and cardiac CT. (A) Twelve-lead ECG showed non-sustained Ventricular Tachycardia and frequent premature ventricular contractions; (B) Three-dimensional caridac enhanced CT revealed pulmonary vein (left panel, red arrowhead) anomaly drainaged into superior vena cava (left panel, blue arrowhead; right panel, red triangle). LA = left atrium, NSVT = non-sustained ventricular tachycardia, PV = pulmonary vein, PVCs = premature ventricular contractions, RA = right atrium, RV = right ventricle, SVC = superior vena cava.

A 57-year-old Chinese man with a history of atrial septal defect (ASD), partial anomalous pulmonary venous drainage (PAPVD) (Fig. 1A) and severe PH was admitted to our center due to paroxysmal palpitation associated with presyncope at rest. Non-sustained ventricular tachycardia (VT) and frequent premature ventricular contractions were recorded by surface electrocardiogram (ECG) when symptoms occurred (Fig. 1B). Echocardiography revealed enlargement of right heart (RV: 43 mm; right atrium: 67 mm), increasement of systolic pulmonary artery pressure (systolic pulmonary artery pressure [SPAP]: 88 mm Hg) and normal ejection fraction (55%). Additionally, he also suffered from obvious dyspnea when climbing stairs World Health Organization Class III (WHO Class III). Catheter closure of ASD was performed 5 years ago but he refused to undergo operation for PAPVD due to worry of open surgery complications.

For long-term management of VAs, antiarrhythmic drugs (AADs) were considered. However, it was worrying that the negative inotropic effect of most AADs may exacerbate his cardiac dysfunction and undermine his exercise tolerance. Amiodarone is comparatively less negatively inotropic. But this patient also presented prolonged QT (440 ms) and QTc interval (445 ms), which suggested he had a high risk of suffering from lethal malignant VT. Thus, amiodarone was relatively contraindicated due to its adverse effect on significantly prolonging QT interval.

**Figure 2:**
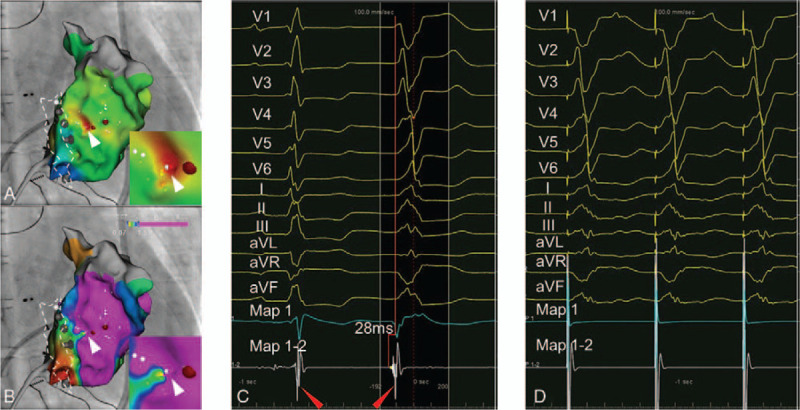
(A and B) Right anterior oblique view of activation mapping and voltage mapping demonstrated the EAS (white triangle) located close to a small low-voltage region at mid free wall of RV near tricuspid annulus (white dash line). (C) Local activation at the EAS significantly preceded surface ECG (28 ms) and presented QS pattern on unipolar electrode and reversed potentials (red triangle) on bipolar electrode. (D) Perfect pace mapping at the EAS underpinned it as site of origin. EAS = earlies activation site.

After informed consent was obtained, we performed EPS and RFA. The procedure was performed under conscious without sedation. Local anesthesia was achieved with lidocaine. With activation mapping, we captured the earliest activation site (EAS) locating very close to a small low-voltage area at the lateral wall of RV near tricuspid annulus, presenting as a focal activation preceding surface ECG by 28 ms (Fig. 2A, B and C). Furthermore, QS pattern on unipolar electrode, reversed potentials on bipolar electrode and perfect pace mapping at this site underpinned it as site of origin (SOO) (Fig. 2C and D). RFA was performed in power-controlled mode with the maximum temperature of 50°C and the maximum power was 30W. Fortunately, radiofrequency delivery at the EAS terminated VAs successfully, which confirmed it as SOO. During the operation and hospital stay, the patient did not have any complication occurred.

During 6 months of follow-up, no VAs relapsed. Echocardiography did not reveal any significant changes in RV dimension and LV function except the dramatical increasement of SPAP (139 mm Hg). Contrary to the exacerbation of PH, the patient got an improvement in exercise tolerance, just with mild dyspnea when climbing stairs World Health Organization Class II (WHO class II).

## 3 Discussion

Hemodynamically-instable VAs in patients with PH are rare.^[2]^ Additionally, information about the substrate, mechanism, electrophysiological characteristics, therapy and prognosis of VAs is lacking.

Previous experimental studies demonstrated that RV hypertrophy and increased myocardial fibrosis in rats with PH generated the substrate for the initiation and maintenance of VAs.^[3–5]^ Cardiomyocytes from failed RV exhibited prolonged action potential duration, early afterdepolarizations, trigger activity, and spontaneous VAs.^[3]^ These laboratory findings revealed the mechanism that RV remodeling secondary to PH initiated VAs.

To the best of our knowledge, only 1 case concerning the mechanism and electrophysiological characteristics of VAs associated with PH has been reported.^[1]^ In the previous case, patient suffered from sustained and non-sustained VAs because of the increased automaticity of right Purkinje arborization and EPS of that patient did not find any low-voltage zone in RV.^[1]^ In our case, the ASD and PAPVD were unlike the direct cause of VAs, because these malformations were limited in atrium. Different from the previous case, VAs of our patient just presented as a nonsustained focal activation and its origin was very close to the small low-voltage zone of RV, suggesting the structural remodeling of RV caused by the increasement of preload (left-to-right shunt) and afterload (PH) serves as the substrate of VAs. Therefore, the putative mechanism of VAs in our case is more consistent with the findings of previous animal studies that remodeled RV serves as the substrate for the initiation of VAs.^[3–5]^

For this patient, frequent VAs exacerbated impaired cardiac function and resulted in hemodynamically-instable symptoms. Thus, control of VAs must be the first choice. However, AADs may worsen cardiac function of this patient due to their negative inotropic effect. Besides, the side effect of AADs on prolonging QT interval increases patients’ risk of deteriorating into life-threatening VAs, especially in severe PH patients who present prolonged QT interval due to RV hypertrophy.^[6,7]^ Thus, AADs were not ideal in most severe PH patients.

Finally, we successfully terminated VAs with RFA. During the operation, we observed the VAs in this patient was a focal activation and the SOO can be found with conventional mapping strategies, like capturing the EAS, QS patter on unipolar electrode and reversed potentials on bipolar electrode as well as pace mapping (Fig. 2C and D). Additionally, the previous case demonstrated mapping Purkinje potential may also facilitate finding potential target in some patients.^[3]^

Previous studies demonstrated arrhythmias, including supraventricular and VAs, were strongly associated with death in PH patients.^[8,9]^ The probable reasons concerning this issue mainly include 2 aspects: firstly, patients with PH associated with arrhythmias are prone to have worse RV function, exhibiting lower TAPSE,^[8,10]^ which was associated with a significantly lower risk of death or clinical worsening.^[11]^ Additionally, the most probable cause is arrhythmias can cause rapid deterioration of RV function and a drop in cardiac output. Thus, the patient got an improvement in exercise capacity (from WHO class III to WHO class II) post operation, despite the exacerbation of PH (from 88 mm Hg–139 mm Hg) due to the uncorrected PAPVD. During follow-up, VAs in both our patient and the previous patient have not relapsed, suggesting favorable short-term efficacy of catheter ablation.

Our case firstly demonstrated RV remodeling, low-voltage zone in this case, serves as the substrate of VAs in patients with PH and RFA may be superior to AADs for them to terminate VAs. The termination of VAs can significantly improve the patient's exercise tolerance despite the exacerbation of PH.

## Author contributions

**Conceptualization:** Song Zou.

**Data curation:** Song Zou, Zhifu Cen, Ruikun Jia.

**Formal analysis:** Song Zou, Zhifu Cen, Ruikun Jia, Sijie Lu.

**Investigation:** Song Zou, Zhifu Cen, Sijie Lu, Yan Hao.

**Methodology:** Song Zou, Yan Hao.

**Software:** Song Zou, Yan Hao.

**Supervision:** Kaijun Cui.

**Validation:** Kaijun Cui.

**Visualization:** Yan Hao, Kaijun Cui.

**Writing – original draft:** Song Zou.
